# Ultrafast Recovery of Uranium from Seawater by *Bacillus velezensis* Strain UUS‐1 with Innate Anti‐Biofouling Activity

**DOI:** 10.1002/advs.201900961

**Published:** 2019-07-24

**Authors:** Yihui Yuan, Qiuhan Yu, Shuo Yang, Jun Wen, Zhanhu Guo, Xiaolin Wang, Ning Wang

**Affiliations:** ^1^ State Key Laboratory of Marine Resource Utilization in South China Sea Hainan University Haikou 570228 P. R. China; ^2^ Institute of Nuclear Physics and Chemistry China Academy of Engineering Physics Mianyang 621900 P. R. China; ^3^ Integrated Composites Laboratory (ICL) Department of Chemical and Biomolecular Engineering University of Tennessee Knoxville TN 37996 USA; ^4^ College of Chemical and Environmental Engineering Shandong University of Science and Technology Qingdao 266590 P. R. China

**Keywords:** anti‐biofouling, bacterium, marine bacteria, seawater, uranium recovery

## Abstract

Highly‐efficient recovery of uranium from seawater is of great concern in the growing demand for nuclear energy. Bacteria are thought to be potential alternatives for uranium recovery. Herein, a *Bacillus velezensis* strain, UUS‐1, with highly‐efficient uranium immobilization capacity is isolated and is used in the recovery of uranium from seawater. The strain exhibits time‐dependent uranium recovery capacity and only immobilizes uranium after growing for 12 h. The carboxyl group together with the amino group inside the bacterial cells, but not previously identified phosphate group, are essential for uranium immobilization. UUS‐1 shows broad‐spectrum antimicrobial activity by producing diverse antimicrobial metabolites, which endows the strain with innate resistance to the biofouling of marine microorganisms. Based on the dry weight of the initially used bacterial cultures, UUS‐1 concentrates uranium by 6.26 × 10^5^ times and reaches the high immobilization capacity of 9.46 ± 0.39 mg U g^−1^ bacterial cultures in real seawater within 48 h, which is the fastest uranium immobilization capacity observed from real seawater. Overall considering the ultrafast and highly‐efficient uranium recovery capacity and the innate anti‐biofouling activity, UUS‐1 is a promising alternative for uranium recovery from seawater.

## 1 Introduction

The growing demand for nuclear energy in recent years has created an urgency for exploring a new supply of uranium.^1^ The ocean, which contains ≈4.5 billion tons of uranium, is thought to be a promising supply, while the extremely low uranium concentration (3.3 ppb, parts per billion) necessitates the development of a highly efficient strategy for uranium recovery. Furthermore, as a type of radioactive heavy metal, the pollution from uranium presents hazards to the environment and to public health.^2^ Therefore, the immobilization of uranium from a low‐concentration uranium‐containing environment and the remediation of uranium from uranium‐contaminated wastewater that would present hazards to public health are of great concerns for researchers all over the world.^3^ For uranium sequestration, many strategies, including adsorption, precipitation, ion exchange, and biological recovery of uranium, have been developed.^4^ Currently, the recovery and remediation of uranium are mainly based on the sorption of uranium ions by using adsorbents.^1^ The organic and inorganic adsorbents show high potential for the removal of uranium. However, due to the high costs of these methods, the discovery of a novel uranium recovery technology is urgently needed.^5^

Immobilization of uranium by using biological entities seems to be a potential alternative due to their fast growth, low cost, and environmentally friendly features. Several biological entities were proven to have uranium sorption capacity,^6^ including plants,^7^ algae,^8^ fungi,^9^ and bacteria.^10^ Among them, the bacteria exhibit the highest potential due to their fast growth and low requirement‐growth conditions. The bacteria contain diverse active groups for uranium adsorption and show a divergent mechanism in uranium immobilization. The phosphate and organic carboxyl functional groups on the microbial surfaces are responsible for the precipitation of uranium U(VI) by forming insoluble uranyl‐phosphate minerals, such as autunite [Ca(UO_2_)_2_(PO_4_)_2_], chernikovite [HUO_2_PO_4_], and uramphite [(NH_4_)(UO_2_)PO_4_·3H_2_O].^11^ Several microbes further show a reduction capacity for uranium by converting mobile U(VI) to more stable U(IV) by forming U(IV)‐oxide minerals (uraninite, UO_2_) or nonuraninite U(IV)‐phosphate minerals, such as CaU(PO_4_)_2_·H_2_O and U_2_O(PO_4_)_2_.^12^ Moreover, some bacteria encoding proteins, such as the highly expressed S‐layer protein from *Pelosinus* sp.^13^ and a protein encoded by *Methanobacterium thermoautotrophicum* that was genetically modified to a highly selective uranium binding protein SUP,[qv: 4f] were reported to maintain uranium immobilization capacities. According to previous reports, microbes showed high uranium recovery capacity from a uranium‐spiked medium, while there is no report on the function of microbes in recovering uranium from high salinity environments, including seawater.

During the sorption of uranium in seawater, the biofouling of microorganisms can present severe hazards to the uranium recovery capacity.^14^ The development of sorption materials that can inhibit the binding of biological entities is crucial for the practical application of adsorbents. However, there are only a few studies on the construction of anti‐biofouling adsorbents.^15^ With coexisting microbes, the microbes compete with each other by competing for nutrients or by synthesizing antimicrobial substances.^16^ Thus, the use of microbes with both uranium immobilization capacity and antimicrobial activity would be a promising strategy for uranium recovery from seawater.

**Figure 1:**
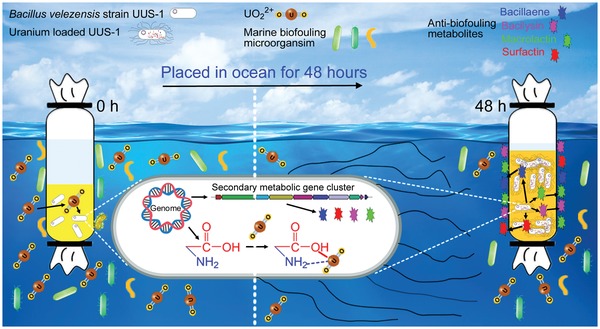
Scheme of the uranium uptake mechanism by *Bacillus velezensis* strain UUS‐1 from seawater.

In this study, a *Bacillus velezensis* strain UUS‐1 that exhibited ultrafast and high uranium uptake capacity, high salt tolerance, high uranium tolerance, and broad antimicrobial activity by producing antimicrobial metabolites, was screened. The bacterium showed high uranium recovery capacity both in uranium‐spiked medium and in the real seawater environment. The uranium uptake capacities of the well‐cultured bacterial cells and cell debris were also analyzed, and the functional group used for uranium adsorption was determined. The broad environmental tolerance, high uranium uptake capacity, and innated anti‐biofouling activity provided the strain with high potential for practical application in uranium recovery from seawater (**Figure** **1**).

## 2 Results and Discussion

### 2.1 Screening of Bacteria with Uranium Adsorption Capacity

For the purpose of screening bacteria that could immobilize uranium, the strains stored or isolated by our lab were cultivated in uranium spiked Luria‐Bertani (LB) broth medium with a uranium concentration of 10 ppm (parts per million, 10 mg L^−1^ uranium element) by the addition of uranyl nitrate. The concentration of the residual uranium in the medium was determined after cultivation for 24 h. The results showed that 6 of the 17 tested strains, including one *B. velezensis* strain, one *B. subtilis* strain, one *B. thuringiensis* strain, two *Escherichia coli* strains, and one *Erwinia carotovora* strain, could immobilize more than 50% of the uranium in the medium (Figure S1, Supporting Information). Among them, the *B. velezensis* strain UUS‐1 showed the highest uranium immobilization capacity. Several *Bacillus* strains were reported to have uranium immobilization ability, including strains from the species of *B. cereus*,^17^ *B. thuringiensis*,^18^ *B. subtilis*,^19^ *B. vallismortis*,[qv: 10c] *B. licheniformis*,^20^ and *B. mucilaginosus*,[qv: 11b] and had been used for the preconcentration of uranium,^21^ suggesting that the *Bacillus* strains showed wide‐ranging existing activity for uranium recovery. Due to the close relationship of these strains in evolution, they have similar genetic backgrounds, which can guide the synthesis of active substance for uranium binding and lead to the wide‐existing activity for uranium recovery. However, none of these strains have been used for uranium recovery from seawater. The strain UUS‐1 was isolated from sands at the ocean beach of Haikou City, Hainan Province and was identified as a *B. velezensis* based on the 16S rDNA sequence (GenBank Accession No. MK092976.1) (Figure S2, Supporting Information). The environment from where this strain was isolated indicated that it might be used for uranium recovery from seawater.

### 2.2 Uranium Uptake Capacity

#### 2.2.1 Uranium Immobilization Capacity during Cultivation in Uranium‐Spiked Medium

**Figure 2:**
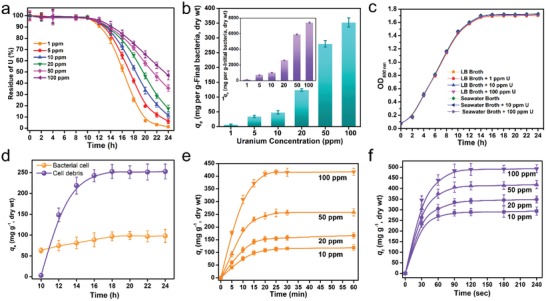
Uranium adsorption capacity and environmental tolerance of UUS‐1. a) Uranium immobilization curve during cultivation. b) Uranium uptake capacity after cultivation for 24 h. The inset item indicates the uranium uptake capacity by calculating the dry weight of the initially added bacterial cultures. c) Uranium and salinity tolerance. d) Time‐dependent uranium uptake capacity of bacterial cells and cell debris. e,f) Adsorption kinetics of bacterial cells and cell debris in uranium‐spiked simulated seawater.

The uranium uptake capacities of UUS‐1 in uranium‐spiked LB broth were determined. After growing in 10 ppm uranium‐spiked LB broth for 24 h, 92.04 ± 1.97% of the uranium was immobilized by strain UUS‐1, and the final uranium immobilization capacity of strain was 48.25 ± 5.61 mg U g^−1^ dry bacterial cells (**Figure** **2**a). Accompanied with the increase of uranium concentration in the medium, the uranium uptake capacity was increased and reached up to 352.34 ± 15.31 mg U g^−1^ dry bacterial cells for UUS‐1 cultivated in 100 ppm uranium‐spiked LB broth for 24 h. In this study, UUS‐1 was inoculated in uranium‐spiked LB broth by adding the exponential bacterial cells into the fresh medium at a ratio of 1:100 (V/V), and the dry weight of the bacterial cells in the medium was increased by 187.52 times after growing for 24 h. Thus, by using the dry weight of the initially added bacterium together with the dry weight of the initially added media (15 g L^−1^), the final uranium uptake capacity was 20.8 times higher, which reached up to 7427.28 ± 68.29 mg U g^−1^ dry bacterial cells for strains cultivated in 100 ppm uranium‐spiked LB broth medium (Figure 2b). The growth ability and the high uranium immobilization capacity could significantly reduce the amount of initially used bacteria, which could further reduce the economic cost for uranium recovery from seawater.

#### 2.2.2 Salinity and Uranium Tolerance

The salinity tolerance of the bacterium is critical for the application of the bacterium in a high salinity environment.^22^ Although several bacteria and fungi were reported to exhibit the ability for uranium immobilization, they are not resistant to seawater salinity or their tolerance to seawater salinity is unknown. The bacterial strain UUS‐1 was isolated from an ocean beach and showed high salinity tolerance. By replacing the LB broth with seawater broth, with a salinity increased from 1.0 to 3.5%, the strain only showed no significant decrease in its growth curve (Figure 2c). As a type of toxic heavy metal, uranium was reported to be toxic to several microorganisms. However, even when cultivated in media containing 100 ppm uranium, which is 3.03 × 10^4^ times the concentration in seawater, the strain showed no significant change in its growth curve. The high tolerance to salinity and uranium endowed the strain with high potential for use in the recovery of uranium from different types of seawater and uranium‐contaminated wastewater.

#### 2.2.3 Uranium Immobilization Capacities of Bacterial Cell and Cell Debris

During the cultivation of strain UUS‐1 in uranium‐spiked medium, the strain only showed uranium uptake capacity after growing for 12 h (Figure 2a), indicating that the uranium uptake capacity of UUS‐1 is dependent on the growth stage, which might be because the synthesis of the active compounds involved in uranium adsorption occurred after 12 h of growth. To determine the potential functional components for uranium immobilization, bacterial cells of different growth stages were collected, and cell debris was prepared by ultrasonication. The uranium uptake capacity was determined by soaking bacterial cells/cell debris with a dry weight of 5 mg in 500 mL 10 ppm uranium‐spiked simulated seawater, which comprised of 438.607 × 10^−3^ m sodium chloride and 2.297 × 10^−3^ m sodium bicarbonate in ultrapure water, for 30 min. The result showed that the bacterial cells only exhibited slight increases in uranium immobilization capacity along with the extension of growth time and reached the highest uranium immobilization capacity of 97.09 ± 13.25 mg U g^−1^ dry bacterial cell of 18 h growth (Figure 2d). However, the cell debris showed significantly increased uranium uptake capacity accompanied by the extension of growth time and reached the highest uranium uptake capacity of 252.54 ± 17.97 mg U g^−1^ dry cell debris of 18 h growth. Furthermore, the uptake of uranium was only observed for the cell debris prepared with more than 12 h growth bacterial cells, which corresponds with the uranium immobilization capacity of the bacterium during growth. The 10 h growth bacterial cells only showed a low uranium uptake capacity of 63.27 ± 6.31 mg U g^−1^ dry bacterial cells, which might be caused by the nonspecific adsorption of uranium by a functional group outside of the bacterial cell, such as a carboxyl group and amino group. Considering the time‐dependent uranium immobilization capacity and the lower uranium uptake capacity of bacterial cells compared with cell debris, it is rational to speculate that UUS‐1 immobilized uranium via the time‐dependent production of functional components inside the bacterial cell.

The uranium uptake capacities of the bacterial cell and cell debris were also analyzed using an 18 h growth bacterium. The results showed that the bacterial cells reached equilibrium adsorption capacities of 108.11 ± 7.25, 152.83 ± 10.24, 243.87 ± 12.37, and 410.18 ± 10.19 mg U g^−1^ dry bacterial cell in 10, 20, 50, and 100 ppm uranium‐spiked simulated seawater, respectively. According to previous reports, compared with the other bacteria with uranium immobilization capacity, UUS‐1 showed the highest uranium uptake capacity in a 100 ppm uranium solution.[qv: 10c,11a,18,23] The bacterial cell debris showed increased uranium uptake capacity and reached equilibrium adsorption capacities of 285.64 ± 14.73, 335.13 ± 17.18, 406.25 ± 24.63, and 483.31 ± 31.34 mg U g^−1^ dry bacterial cell debris in 10, 20, 50, and 100 ppm, uranium‐spiked simulated seawater, respectively. The cell debris showed significantly higher uranium uptake capacity in uranium‐spiked simulated seawater with a low uranium concentration, which might be because the cell debris contained fewer substances that were not essential with uranium immobilization, such as soluble DNA, polysaccharides, and nonfunctional cellular contents. The ultrasonic process caused the collapse of the bacterial cells, and these types of soluble nonessential compounds were released into the supernatant after centrifugation. In addition, the cell debris showed an extremely fast equilibrium time and reached a saturated adsorption capacity within 90 s, while the bacterial cells reached an adsorption equilibrium in 20 min. We speculate that this might be because the ultrasonication of the cell released the functional components for the uranium binding and increased the contact between uranium and the functional group for uranium adsorption.

### 2.3 Mechanism of Uranium Immobilization

#### 2.3.1 Identification of Functional Groups for Uranium Immobilization

**Figure 3:**
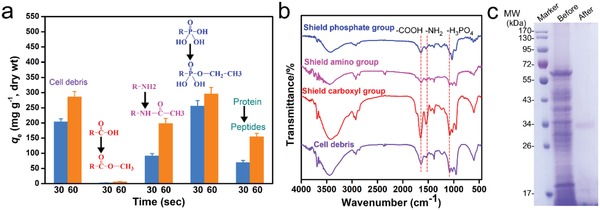
Identification of functional components for uranium immobilization. a) Uranium uptake capacity. b) FTIR spectra. c) Protein profiles of cell debris before and after proteinase K treatment.

To identify functional groups that were used by UUS‐1 for uranium immobilization, the cell debris of 18 h‐growth UUS‐1 was prepared. The carboxyl group, the amino group, the phosphate group, and the proteins were shielded by carboxyl esterification (CH‐E), aminoacetylation (NH‐A), phosphorous esterification (PO‐E), and proteinase K treatment, respectively, and the group‐shielded cell debris was used for uranium extraction in 10 ppm uranium‐spiked simulated seawater. The analysis of uranium uptake capacity showed that the shielding of the carboxyl group caused the loss of uranium adsorption capacity, the shielding of the amino group and the treatment of proteinase K caused significant decrease of uranium adsorption capacity, while the shielding of the phosphate group had no influence on uranium adsorption capacity (**Figure** **3**a). This result suggested that the carboxyl group was essential for the uranium uptake capacity and that the amino group could facilitate the uranium uptake process, while the phosphate group did not take part in the uranium uptake process. According to previous reports, *Bacillus* strains immobilized uranium mainly by the interaction of phosphate with uranyl in the solution and formed uranyl‐phosphate minerals. The results of this study revealed that the strain used in this study maintained a novel mechanism for uranium immobilization. The group‐shielded cell debris was analyzed by Fourier transform infrared spectroscopy (FTIR), and the results indicated that these reactions were all successful (Figure 3b). The peak of the carboxyl group showed a shift from 1640 to 1652 cm^−1^ for the carboxyl group‐shielded cell debris. The peaks of the amino group (peak at ≈1543 cm^−1^) and phosphate group (peak at ≈1079 cm^−1^) were weakened in the amino group and phosphate group‐shielded cell debris, respectively. The carboxyl group and the amino group constituted an amino, which was the basic building block of proteins. To identify whether it was a protein that immobilized uranium, the cell debris was treated with proteinase K to remove the proteins. After being treated with proteinase K for 12 h, the protein in the cell debris was reduced significantly, and the cell debris only maintained 56.31% of its initial dry weight. As shown in the protein profile of the cell debris treated with proteinase K, the bands of most of the proteins disappeared, suggesting that protein might be the functional component for uranium immobilization (Figure 3c).

#### 2.3.2 Characterization of Uranium‐Loaded Bacterial Cells

**Figure 4:**
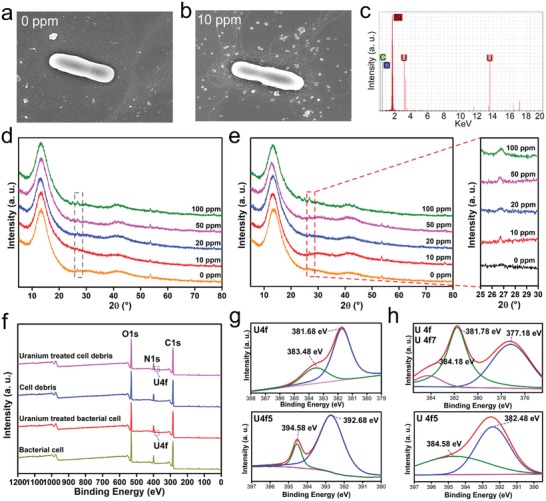
Characterization of the uranium‐loaded bacterial cell. a,b) SEM image of UUS‐1 cultured in uranium‐free medium and uranium‐spiked medium. c) EDS spectra of uranium‐loaded bacterial cells. d,e) XRD patterns of bacterial cells and bacterial cell debris. f) XPS spectra of bacterial cells and ultrasonicated bacterial cells. g,h) High‐resolution XPS spectra of uranium in bacterial cells and cell debris.

The bacteria cultivated in uranium‐spiked seawater broth were observed by scanning electron microscopy (SEM). The results showed that the addition of 10 ppm uranium could change the morphology of bacterial cells and caused the formation of more flagella (**Figure** **4**a,b). The flagellum is essential for the movement of the bacterium to a suitable environment for growth. As a heavy metal, a high concentration of uranium was possibly toxic to biological entities, and UUS‐1 formed more flagella to escape the high uranium environment. The energy‐dispersive spectrometry (EDS) spectra analysis showed that uranium was observed on the bacterial cell, which proved the immobilization of uranium by UUS‐1 (Figure 4c). The uranium‐loaded bacterial cell and cell debris were also analyzed by X‐ray diffraction (XRD) and the results showed that additional peaks were observed in the XRD spectra of uranium‐treated bacterial cells and cell debris, which indicated that new crystal structure was formed (Figure 4e). The uranium‐loaded bacteria were also analyzed using X‐ray photoelectron spectroscopy (XPS). After cultivation in uranium‐spiked seawater broth, the peaks of uranium appeared at 377.08 and 393.08 eV, which corresponded to the peak of U4f and the valence state of uranium in uranyl nitrate (VI), suggesting that there is no redox reaction during the immobilization process of uranium (Figure 4f). The high‐resolution XPS spectra of uranium‐treated bacterial cells and cell debris corresponded with these results (Figure 4g,h). Several bacterial strains showed uranium immobilization activity by chemical reduction of uranium (VI) to less soluble uranium (IV).^12^ The findings of this study suggested that UUS‐1 immobilized uranium involved a different strategy.

### 2.4 Antimicrobial Activity

**Figure 5:**
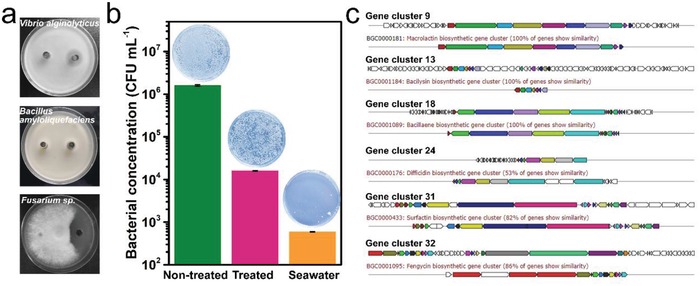
Antimicrobial activity and gene clusters for antimicrobial compound synthesis of UUS‐1. a) Antimicrobial activities against marine microorganisms. b) Inhibition on the growth of marine bacteria. c) Predicted gene clusters responsible for the synthesis of antimicrobial substances.

Several bacteria belonging to the *B. subtilis* group were reported to have the ability of producing antimicrobial substances.^24^ Antimicrobial activity analysis showed that the fermentation liquid produced by UUS‐1 could inhibit the growth of several tested marine microbiomes, including the Gram positive bacterium *B. amyloliquefaciens*, the Gram negative bacterium *Vibrio alginolyticus*, and the fungus *Fusarium* sp. (**Figure** **5**a). The antibacterial effects of the fermentation liquid on the growth of the marine microorganism community were also determined. By cocultivating the seawater with the fermentation liquid of UUS‐1 in seawater broth, the addition of 2% fermentation liquid showed high inhibition to the growth of the marine microorganisms. After cocultivation for 6 h, the bacterial concentration of the cultures that were treated and not treated with fermentation liquid reached 1.59 × 10^4^ and 1.60 × 10^6^ CFU mL^−1^, respectively, indicating that the fermentation liquid inhibited 99.01% of the growth of the marine bacteria (Figure 5b).

To analyze the potential antimicrobial substance produced by UUS‐1, the genome of UUS‐1 was sequenced (GenBank Accession No. SNVA00000000) (Figure S3, Supporting Information), and the antimicrobial compound synthesis associated gene clusters were identified using antiSMASH 4.0 (Figure S4, Supporting Information). The results showed that 100% of UUS‐1 genes showed similarity with gene clusters responsible for the synthesis of several antimicrobial substances (Figure 5c; Table S1, Supporting Information), including macrolactin, bacilysin, and bacillaene (Figure S5, Supporting Information), which all showed antibacterial activity.^25^ Moreover, the genome of the strain also contained more than 50% of genes with similarity to the gene clusters involved in synthesis of difficidin, surfactin, and fengycin, which showed antibacterial or antifungal activity.[qv: 25a,26] The presence of the gene cluster responsible for the synthesis of secondary metabolites with antibacterial and antifungal activity corresponded with the broad antimicrobial spectrum of UUS‐1, which could benefit the use of the strain in the ocean environment by inhibiting biofouling.

### 2.5 Accumulation of Uranium in Natural Seawater

**Figure 6:**
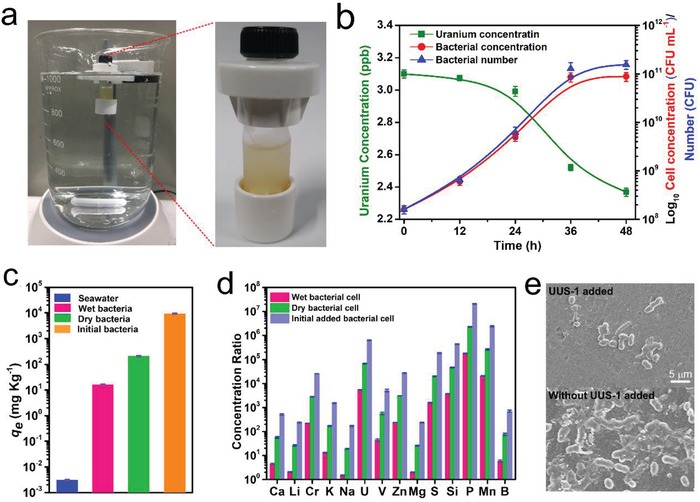
Uranium uptake in natural seawater. a) Equipment used for testing uranium uptake capacity in natural seawater. b) Bacterial growth curve and kinetics curve for uranium uptake. c) Uranium uptake capacity. d) Concentration ratio of elements from seawater. e) SEM observation of the outside of the dialysis tube after uranium extraction in seawater.

The uranium accumulation ability of UUS‐1 in real seawater was determined using the equipment shown in **Figure** **6**a. The exponential growth phase UUS‐1 was added into the dialysis tube together with LB broth for bacterial growth. A dialysis tube with a retention size of 3 kDa was used, which not only limited the release of nutrients from the dialysis tube and the entrance of marine microorganisms but also released the antimicrobial compounds produced by UUS‐1. In seawater, UUS‐1 showed uranium uptake capacity after growing for 24 h, which was slow compared with bacteria cultivated in seawater broth (Figure 6b). This might be because of the lack of nutrients. Although the retention size of the dialysis tube used was relatively small and capable of retaining the added peptone and yeast extract in the LB broth, but some small molecular nutrients were released into the seawater. After growth in seawater for 48 h, UUS‐1 immobilized 24.33% of the uranium in 1 L of seawater, and the concentration of UUS‐1 cells increased by ≈555.63 times, while the total number of UUS‐1 increased by 975.26 times, which was because the volume of bacterial suspension in the dialysis tube increased by 1.76 times. Due to the growth of the bacterium, the osmotic pressure in the dialysis tube increased, and seawater was absorbed into the dialysis tube. Compared with the concentration of uranium in seawater, uranium in the bacterium was concentrated by 5329.02 ± 245.36 times and 68 996.77 ± 3176.77 times, respectively, based on the weight of wet bacterial cells and dry bacterial cells (Figure 6c). The uranium uptake capacity of the bacterium reached 16.52 ± 0.70 and 213.89 ± 9.01 mg kg^−1^, respectively, by calculating the final weight of the wet bacterial cells and dry bacterial cells. Due to the growth of UUS‐1, by calculating the dry weight of the initially added bacterial cells and the initially used media (40 mg mL^−1^), the uranium in seawater was concentrated by 6.26 × 10^5^ times and reached a uranium immobilization capacity of up to 9.46 ± 0.39 mg U g^−1^ of initially added cultures. The ability of UUS‐1 to immobilize other elements has also been determined. The result showed that, except for uranium, UUS‐1 also showed high uptake capacity for other elements, such as P, S, Mn, Fe, and Na (Figure 6d), which was because that these elements were essential for the growth of bacteria by taking part in their life process. Some other elements, including Cu, Ni, Zn, and Si, were also highly concentrated, which might be adsorbed by the nonspecific binding of the bacterium (Table S2, Supporting Information). In consideration of the antimicrobial capacity, the adhesion of microorganisms onto the outside of the dialysis tube was observed by SEM. Compared with the dialysis tube without adding of UUS‐1, only a few microorganisms adhered to the dialysis tube containing UUS‐1, suggesting that the antimicrobial compounds produced by UUS‐1 could inhibit the adhesion of marine microorganisms (Figure 6e).

## 3 Conclusion

In conclusion, this study for the first time isolated a bacterial strain with ultrafast and high uranium immobilization capacity as well as innate anti‐biofouling activity from real seawater. In real seawater, based on the dry weight of the initially used bacterial cultures, UUS‐1concentrated uranium from seawater by 6.26 × 10^5^ times and reached the uranium immobilization capacity of 9.46 ± 0.39 mg U g^−1^ of initially added cultures within 48 h, which was the fastest uranium immobilization capacity observed from real seawater. The uranium recovery mechanism analysis revealed that the strain exhibited time‐dependent active for uranium immobilization and only immobilized uranium after growing for 12 h. The carboxyl group together with the amino group inside the bacterial cells, but not previously identified phosphate group, were essential for uranium immobilization. The genome of the strain contained the gene clusters for several antimicrobial metabolites, which endowed the strain with innate resistance to biofouling of marine microorganisms. Overall considering the high and ultrafast uranium immobilization capacity, the innate resistance to the biofouling of marine microorganisms, and the low cost for preparation, strain UUS‐1 is a promising alternative for uranium recovery from ocean.

## 4 Experimental Section

*Screening and Identification of Uranium‐Immobilizing Bacteria*: To screen for strains that could immobilize uranium, the exponential growth strains were inoculated into LB broth, which was prepared with tryptone (10 g L^−1^), yeast extract (5 g L^−1^), and NaCl (10 g L^−1^) and autoclaving at 121 °C for 20 min. Then filtration sterilized uranyl nitrate was added into the medium to a final concentration of 10 ppm. For all the experiments in this study, the pH of the uranium‐spiked mediums was adjusted to 7.2 by addition of NaOH solution before the incubation of bacterial strains. The strains used in this study were isolated or stored by the lab and are listed in Figure S1 (Supporting Information). After further cultivation at 30 °C in a thermostatic shaker for 24 h with moderate shaking, the supernatants of the cultures were collected by centrifugation (10 000 rpm, 5 min). The cultivations of the bacteria in uranium‐spiked medium were carried out in triplicate. The concentration of uranium in the supernatants was determined by inductively coupled plasma optical emission spectrometry (ICP‐OES) to determine the uranium immobilization capacity. The bacterial strain that showed the highest uranium immobilization capacity was identified using 16S rDNA sequencing, and the species of the strains were determined by phylogenetic analysis using the software MEGA7.^27^ The genomes of the strains were determined from the exponential growth strain using a previously described method, and the genome sequencing was performed using Illumina HiSeq 2500.^28^ The antimicrobial compound biosynthesis gene clusters encoded by the genomes were analyzed using the software antiSMASH 4.0, bacterial version.^29^

*Characterization of the Uranium Immobilization Capacity*: To determine the uranium immobilization capacity of the bacterial strain, the exponential growth strain was inoculated into LB broth with spiked uranyl nitrate to a final uranium concentration of 1, 5, 10, 20, 50, and 100 ppm. The cultures were collected at an interval of 2 h, and the concentration of the soluble uranium in the supernatant was determined using ICP‐OES. For each uranium concentration, three replicates were performed. The dry weight of the cell pellets was determined by drying using a vacuum drying oven at 50 °C until the weight of the cell pellet was constant. The immobilized capacity was determined using Equation (1)

(1) $$q_{t}\;=\;\left(C_{0}\;-\;C_{t}\right)\;\times \;\frac{V}{m}$$

where *q*_t_ (mg U g^−1^ bacterial dry weight) indicates the uranium immobilized at time *t*, *C*_0_ (ppm) indicates the initial uranium concentration, *C*_t_ (ppm) indicates the uranium concentration at time *t*, *V* (L) indicates the volume of the used LB broth, and *m* (g) indicates the dry weight of bacteria at time *t*. The uranium immobilization capacity of the cultured bacterial cells and cell debris was also determined. To prepare cell debris, the bacterial cell pellet of UUS‐1 was collected by centrifugation and ultrasonicated using an ultrasonic cell disruptor JY92‐IIDN. After centrifuging (10 000 rpm, 30 min) at 4 °C, the pellet was collected for uranium uptake capacity assay. Basically, the bacterial cells or cell debris with a dry weight of 10 mg were suspended in 1 L uranium‐spiked seawater of different uranium concentrations with a pH of 7.0 and shaken at 30 °C by using a thermostatic shaker throughout the test, and the uranium uptake capacity was calculated using Equation (1). All experiments were performed in triplicate.

*Assay of Environmental Tolerance*: The tolerance of the bacterium to seawater and uranium was determined by analyzing the growth curve of the bacterium in uranium‐spiked seawater broth. The seawater broth was prepared by adding tryptone (10 g L^−1^) and yeast extract (5 g L^−1^) into seawater and autoclaving at 121 °C for 20 min. The uranyl nitrate was added into the seawater broth to a final concentration of 10, 50, and 100 ppm, and the exponential growth phase of the bacterium was inoculated into the media at a ratio of 1:100 (V/V) and cultivated at 30 °C with moderate shaking (180 rpm). The absorbance of the bacterial cultures was determined at 600 nm at an interval of 2 h using a Multi‐Mode Microplate Reader Synergy HT. All experiments were performed in triplicate.

*Determination of the Uranium Immobilization Mechanism*: To determine the uranium immobilization mechanism of the bacterium, the bacterial cell culture in 100 ppm uranium‐spiked seawater broth was observed using a Hitachi S‐4800 SEM. EDS with a Bruker Nano XFlash Detector 5030 was used to determine the EDS spectra. XRD analysis of the bacteria was performed using a Bruker AXS Diffractometer D8. The FTIR spectra were obtained using a PerkinElmer FTIR spectrometer. A Kratos AXIS‐SUPRA spectrometer was used to analyze the XPS spectra of the bacterium and the bacterial cell debris.

To analyze the functional group used for uranium immobilization, the carboxyl group, amino group, and phosphate group were shielded.[qv: 11a] To shield the carboxyl group, carboxyl esterification (CH‐E) was performed by treating the cell debris with anhydrous methanol (10 mL) and HCl (5.4 mL) with shaking at 30 °C for 3 h. To shield the amino group, aminoacetylation (NH‐A) was performed by treating the cell debris with acetic anhydride/ethanol solution (1:10, V/V, 10 mL) and shaking at 30 °C for 2 h. For purpose of shielding the phosphate group, phosphorus esterification (PO‐E) was carried out by mixing the cell debris with triethyl phosphate/nitromethane solution (4:3, V/V, 14 mL) with shaking at 30 °C for 3 h. To determine the function of proteins in uranium uptake, the cell debris was treated with proteinase K (1 mg mL^−1^) for shielding at 45 °C for 12 h. After washing the above mixture with distilled water for three times, the uranium uptake capacity of the shielded cell debris was determined by three times.

*Assay of Antimicrobial Activity*: The antimicrobial activity of the fermentation liquid of UUS‐1 was determined using the Oxford cup method.^30^ After cultivation in LB broth for 28 h, the bacterial culture was centrifuged (10 000 rpm, 10 min), and the supernatant was collected and further filtered with a 0.22 µm pore‐size membrane filter to remove bacterial cells. After spreading the exponential growth indicator strains onto a solid plate, the Oxford cup was added onto the plate, and fermentation liquid (200 µL) of UUS‐1 was added into the Oxford cup. After cultivation at 30 °C for 18 h, the antimicrobial activity was recorded. The antibacterial activity of the marine bacteria was analyzed by cocultivation of the fermentation with seawater and cocultivation process was performed for three times. The fermentation liquid of UUS‐1 and seawater were added into the seawater broth at a ratio of 1:50 (V/V). After cultivation, the bacterial concentration was determined by dilution counting.

*Uranium Uptake Capacity in Real Seawater*: For uranium uptake from real seawater, 10 µL of the exponential growth phase of UUS‐1 was added into the dialysis tube with a retention size of 3 kDa, as shown in Figure 6a, together with seawater (1 mL seawater), tryptone (25 mg), yeast extract (15 mg), and soaked into real seawater (1 L) with moderate shaking at 30 °C. Three independent experiments were carried out for uranium uptake capacity assay in real seawater. The bacterial concentration in the dialysis tube and the uranium concentration in the seawater were determined every 12 h by using dilution counting and inductively coupled plasma‐mass spectrometry (ICP‐MS), respectively. After cultivation in seawater for 48 h, the bacterial cells in the dialysis tube were collected, and the uptake capacity for uranium and some other elements were determined by ICP‐MS. The outside of the dialysis tube used for this test was observed by SEM.

## Conflict of Interest

The authors declare no conflict of interest.

## Supporting information

Supplementary

## References

[1] C. W. Abney , R. T. Mayes , T. Saito , S. Dai , Chem. Rev. 2017, 117, 13935. 29165997 10.1021/acs.chemrev.7b00355

[2] a) M. Gavrilescu , L. V. Pavel , I. Cretescu , J. Hazard. Mater. 2009, 163, 475; 18771850 10.1016/j.jhazmat.2008.07.103

[3] a) M. M. Aly , M. F. Hamza , J. Dispersion Sci. Technol. 2013, 34, 182;

[4] a) A. S. Ivanov , B. F. Parker , Z. C. Zhang , B. Aguila , Q. Sun , S. Q. Ma , S. Jansone‐Popova , J. Arnold , R. T. Mayes , S. Dai , V. S. Bryantsev , L. F. Rao , I. Popovs , Nat. Commun. 2019, 10, 819; 30778071 10.1038/s41467-019-08758-1 PMC6379418

[5] a) W. Luo , G. Xiao , F. Tian , J. J. Richardson , Y. P. Wang , J. F. Zhou , J. L. Guo , X. P. Liao , B. Shi , Energy Environ. Sci. 2019, 12, 607;

[6] Y. Suzuki , S. D. Kelly , K. M. Kemner , J. F. Banfield , Nature 2002, 419, 134. 12226656 10.1038/419134a

[7] N. Hu , T. Lang , D. X. Ding , J. S. Hu , C. W. Li , H. Zhang , G. Y. Li , J. Environ. Radioact. 2019, 199, 58. 30685639 10.1016/j.jenvrad.2018.12.023

[8] F. Celik , M. A. A. Aslani , S. S. Can , Turk. J. Fish. Aquat. Sci. 2019, 19, 593.

[9] a) C. S. Zhao , J. Liu , H. Tu , F. Z. Li , X. Y. Li , J. J. Yang , J. L. Liao , Y. Y. Yang , N. Liu , Q. Sun , Environ. Sci. Pollut. Res. 2016, 23, 24846; 10.1007/s11356-016-7722-x 27662852

[10] a) S. Choudhary , P. Sar , Rev. Environ. Sci. Bio/Technol. 2015, 14, 347;

[11] a) J. Zhang , H. Song , Z. Chen , S. S. Liu , Y. L. Wei , J. Y. Huang , C. L. Guo , Z. Dang , Z. Lin , Chemosphere 2018, 206, 682; 29783053 10.1016/j.chemosphere.2018.04.181

[12] X. L. Li , C. C. Ding , J. L. Liao , L. Du , Q. Sun , J. J. Yang , Y. Y. Yang , D. Zhang , J. Tang , N. Liu , J. Environ. Sci. 2017, 53, 9. 10.1016/j.jes.2016.01.030 28372765

[13] M. P. Thorgersen , W. A. Lancaster , L. Rajeev , X. X. Ge , B. J. Vaccaro , F. L. Poole , A. P. Arkin , A. Mukhopadhyay , M. W. W. Adamsa , Appl. Environ. Microbiol. 2017, 83, e03044. 27913415 10.1128/AEM.03044-16 PMC5288816

[14] a) J. Park , G. A. Gill , J. E. Strivens , L. J. Kuo , R. T. Jeters , A. Avila , J. R. Wood , N. J. Schlafer , C. J. Janke , E. A. Miller , M. Thomas , R. S. Addleman , G. T. Bonheyo , Ind. Eng. Chem. Res. 2016, 55, 4328;

[15] a) J. Wen , Q. Y. Li , H. Li , M. Chen , S. Hu , H. M. Cheng , Ind. Eng. Chem. Res. 2018, 57, 1826;

[16] a) J. S. Madsen , S. J. Sorensen , M. Burmolle , Curr. Opin. Microbiol. 2018, 42, 104; 29197823 10.1016/j.mib.2017.11.018

[17] J. Zhang , H. Song , Z. Chen , S. Liu , Y. Wei , J. Huang , C. Guo , Z. Dang , Z. Lin , Chemosphere 2018, 206, 682. 29783053 10.1016/j.chemosphere.2018.04.181

[18] X. H. Pan , Z. Chen , F. B. Chen , Y. J. Cheng , Z. Lin , X. Guan , J. Hazard. Mater. 2015, 297, 313. 26026850 10.1016/j.jhazmat.2015.05.019

[19] Y. B. Sun , R. Zhang , C. C. Ding , X. X. Wang , W. C. Cheng , C. L. Chen , X. K. Wang , Geochim. Cosmochim. Acta 2016, 180, 51.

[20] Z. J. Yi , J. Yao , J. Radioanal. Nucl. Chem. 2012, 293, 907.

[21] a) S. Ozdemir , M. K. Oduncu , E. Kilinc , M. Soylak , J. Radioanal. Nucl. Chem. 2018, 315, 185;

[22] a) S. M. Rea , N. J. McSweeney , B. P. Degens , C. Morris , H. M. Siebert , A. H. Kaksonen , Miner. Eng. 2015, 75, 126;

[23] H. Sohbatzadeh , A. R. Keshtkar , J. Safdari , T. Yousefi , F. Fatemi , Chem. Eng. J. 2017, 323, 492.

[24] S. Caulier , C. Nannan , A. Gillis , F. Licciardi , C. Bragard , J. Mahillon , Front. Microbiol. 2019, 10, 302. 30873135 10.3389/fmicb.2019.00302 PMC6401651

[25] a) L. M. Wu , H. J. Wu , L. Chen , X. F. Yu , R. Borriss , X. W. Gao , Sci. Rep. 2015, 5, 12975; 26268540 10.1038/srep12975 PMC4534799

[26] H. Zhao , D. Shao , C. Jiang , J. Shi , Q. Li , Q. Huang , M. S. R. Rajoka , H. Yang , M. Jin , Appl. Microbiol. Biotechnol. 2017, 101, 5951. 28685194 10.1007/s00253-017-8396-0

[27] S. Kumar , G. Stecher , K. Tamura , Mol. Biol. Evol. 2016, 33, 1870. 27004904 10.1093/molbev/msw054 PMC8210823

[28] Y. H. Yuan , M. Y. Gao , Sci. Rep. 2015, 5, 10259. 25989507 10.1038/srep10259 PMC4437294

[29] K. Blin , T. Wolf , M. G. Chevrette , X. W. Lu , C. J. Schwalen , S. A. Kautsar , H. G. S. Duran , E. L. C. de los Santos , H. U. Kim , M. Nave , J. S. Dickschat , D. A. Mitchell , E. Shelest , R. Breitling , E. Takano , S. Y. Lee , T. Weber , M. H. Medema , Nucleic Acids Res. 2017, 45, W36. 28460038 10.1093/nar/gkx319 PMC5570095

[30] Y. X. Fu , L. J. Ma , Y. P. Yi , Y. Fan , J. P. Liang , R. F. Shang , Microb. Pathog. 2019, 127, 202. 30529392 10.1016/j.micpath.2018.12.009

