# Supplementary material for: Ultrafast Recovery of Uranium from Seawater by Bacillus velezensis Strain UUS‐1 with Innate Anti‐Biofouling Activity
Source: Adv Sci (Weinh). 2019 Jul 24;6(18):1900961. doi: 10.1002/advs.201900961 (PMC6755527; doi:10.1002/advs.201900961)
Supplement: Supplementary file 1 — Supplementary [file ADVS-6-1900961-s001.pdf]

Copyright WILEY-VCH Verlag GmbH & Co. KGaA, 69469 Weinheim, Germany, 2019.

ADVANCED SCIENCE
Open Access

Supporting Information

for *Adv. Sci.,* DOI: 10.1002/advs.201900961

Ultrafast Recovery of Uranium from Seawater by *Bacillus velezensis* Strain UUS-1 with Innate Anti-Biofouling Activity

*Yihui Yuan, Qiuhan Yu, Shuo Yang, Jun Wen, Zhanhu Guo, Xiaolin Wang, and Ning Wang**

Copyright WILEY-VCH Verlag GmbH & Co. KGaA, 69469 Weinheim, Germany, 2019.

Supporting Information

Ultrafast Recovery of Uranium from Seawater by *Bacillus velezensis* Strain UUS-1 with Innate Anti-biofouling Activity

*Yihui Yuan, Shuo Yang, Qiuhan Yu, Jun Wen, Zhanhu Guo, Xiaolin Wang, Ning Wang**

Dr. Y. Yuan, S. Yang, Q. Yu, Prof. N. Wang

State Key Laboratory of Marine Resource Utilization in South China Sea

Hainan University

Haikou 570228, P. R. China

Email: wangn02@foxmail.com

Dr. J. Wen, Prof. X. Wang

Institute of Nuclear Physics and Chemistry

China Academy of Engineering Physics

Mianyang 621900, P. R. China

Prof. Z. Guo

Integrated Composites Laboratory (ICL), Department of Chemcial & Biomolecular Engineering

University of Tennessee

Knoxville TN 37996, USA

Figures and Tables

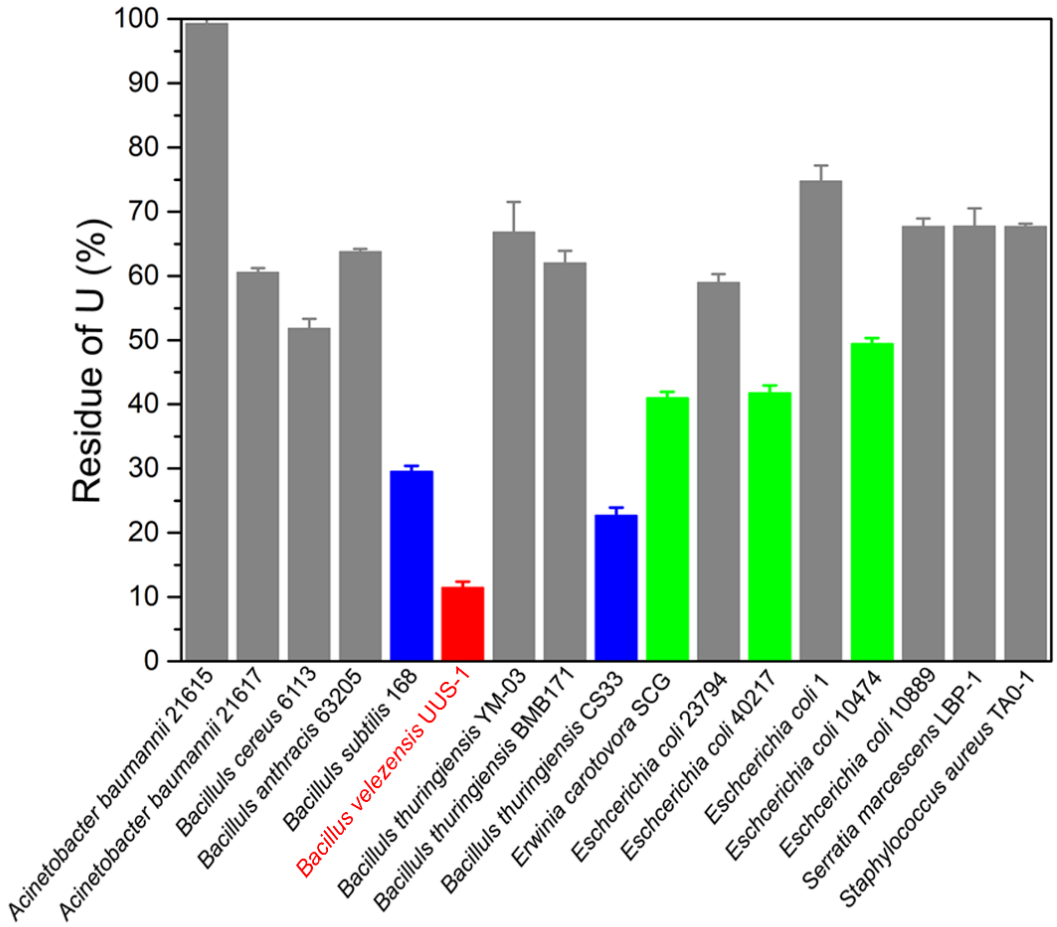

Figure S1. Adsorption capacity of the tested bacteria in uranium recovery. The residual uranium in the medium were determined after growth of the bacteria for 24 h. The initial concentration of the uranium in the medium is 10 ppm.

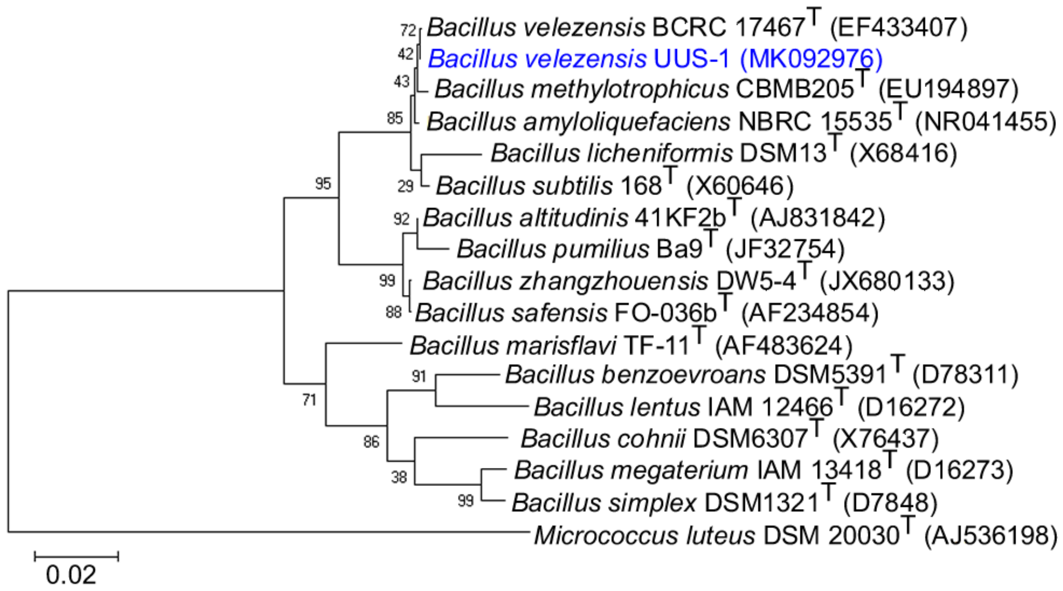

Figure S2. Phylogenetic analysis of *Bacillus velezensis* UUS-1 based on the 16s rDNA sequence.

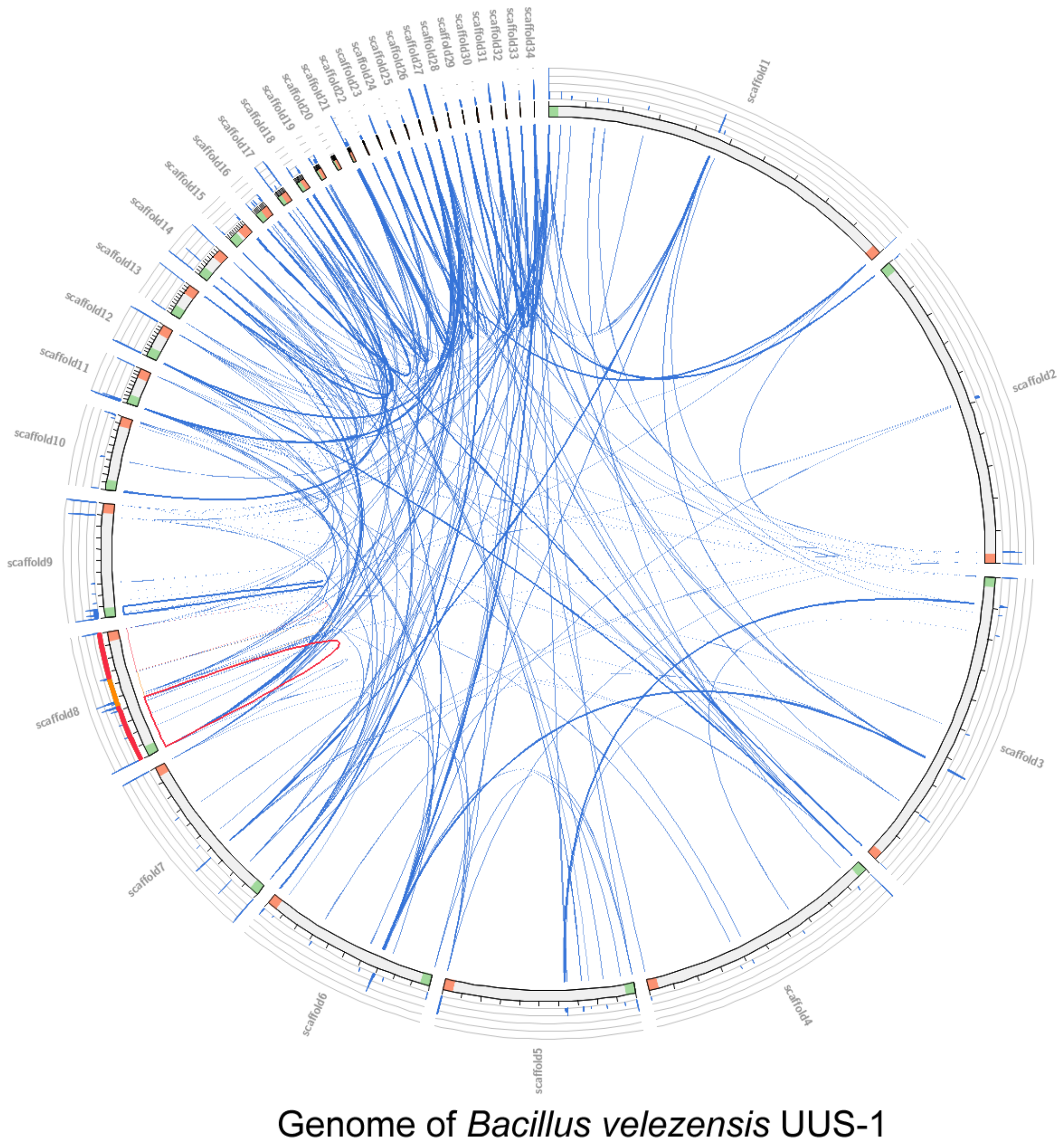

Figure S3. Genome of strain *B. velezensis* strain UUS-1.

Overview 1 2 3 4 5 6 7 8 9 10 11 12 13 14 15 16 17 18 19 20 21 22 23 24 25 26 27 28 29 30 31 32 33 34 35 36 37

Identified secondary metabolite clusters

Cluster	Type	From	To	Most similar known cluster
The following clusters are from record scaffold1:				
Cluster 1	Otherks	65339	106583	Butirosin_biosynthetic_gene_cluster (7% of genes show similarity)
Cluster 2	Cf_fatty_acid	134642	155619	-
Cluster 3	Cf_putative	158186	165321	-
Cluster 4	Terpene	189281	210021	-
Cluster 5	Cf_fatty_acid	221844	246673	Citrulline_biosynthetic_gene_cluster (27% of genes show similarity)
Cluster 6	Cf_putative	267622	276536	-
Cluster 7	Cf_putative	296608	311219	Molybdenum_cofactor_biosynthetic_gene_cluster (11% of genes show similarity)
Cluster 8	Cf_putative	321278	337887	-
Cluster 9	Transatpks	523559	609461	Macrolactin_biosynthetic_gene_cluster (100% of genes show similarity)
The following clusters are from record scaffold2:				
Cluster 10	Nrps	1	40303	-
Cluster 11	Cf_saccharide	43779	99175	Teichuronic_acid_biosynthetic_gene_cluster (100% of genes show similarity)
Cluster 12	Cf_putative	126214	140366	-
Cluster 13	Cf_saccharide	240626	313029	Bacilysin_biosynthetic_gene_cluster (100% of genes show similarity)
Cluster 14	Cf_putative	468358	482046	-
Cluster 15	Cf_saccharide	533124	555507	-
The following clusters are from record scaffold3:				
Cluster 16	Transatpks	504195	533212	Difficidin_biosynthetic_gene_cluster (40% of genes show similarity)
The following clusters are from record scaffold4:				
Cluster 17	Cf_putative	34241	47072	-
Cluster 18	Bacteriocin-Nrps	73617	140413	Bacillibactin_biosynthetic_gene_cluster (100% of genes show similarity)
Cluster 19	Cf_saccharide	322767	348178	-
The following clusters are from record scaffold5:				
Cluster 20	Transatpks-Nrps	24633	127313	Bacillaene_biosynthetic_gene_cluster (100% of genes show similarity)
The following clusters are from record scaffold6:				
Cluster 21	Terpene	7988	29871	-
Cluster 22	Cf_putative	71781	76491	-
Cluster 23	T3pks	110030	151130	-
Cluster 24	Transatpks	265769	311755	Difficidin_biosynthetic_gene_cluster (53% of genes show similarity)
The following clusters are from record scaffold7:				
Cluster 25	Phosphonate	24657	65541	-
Cluster 26	Cf_saccharide	116240	150139	Plantathiazolicin_/_plantazolicin_biosynthetic_gene_cluster (83% of genes show similarity)
Cluster 27	Cf_putative	233029	256728	-
The following clusters are from record scaffold8:				
Cluster 28	Cf_fatty_acid	15215	36450	-
Cluster 29	Cf_putative	122753	129128	-
Cluster 30	Cf_putative	186730	202312	-
The following clusters are from record scaffold9:				
Cluster 31	Nrps	1	55523	Surfactin_biosynthetic_gene_cluster (82% of genes show similarity)
The following clusters are from record scaffold10:				
Cluster 32	Transatpks-Nrps	1	89027	Fengycin_biosynthetic_gene_cluster (86% of genes show similarity)
The following clusters are from record scaffold15:				
Cluster 33	Cf_putative	2667	13703	-
Cluster 34	Nrps	15134	37572	Plipastatin_biosynthetic_gene_cluster (46% of genes show similarity)
The following clusters are from record scaffold16:				
Cluster 35	Transatpks	1	23898	Difficidin_biosynthetic_gene_cluster (26% of genes show similarity)
The following clusters are from record scaffold18:				
Cluster 36	Nrps	1	15152	Plipastatin_biosynthetic_gene_cluster (23% of genes show similarity)
The following clusters are from record scaffold19:				
Cluster 37	Nrps	1	9857	Fengycin_biosynthetic_gene_cluster (20% of genes show similarity)

Figure S4. Secondary metabolite clusters identified in the genome of *B. velezensis* strain UUS-1.

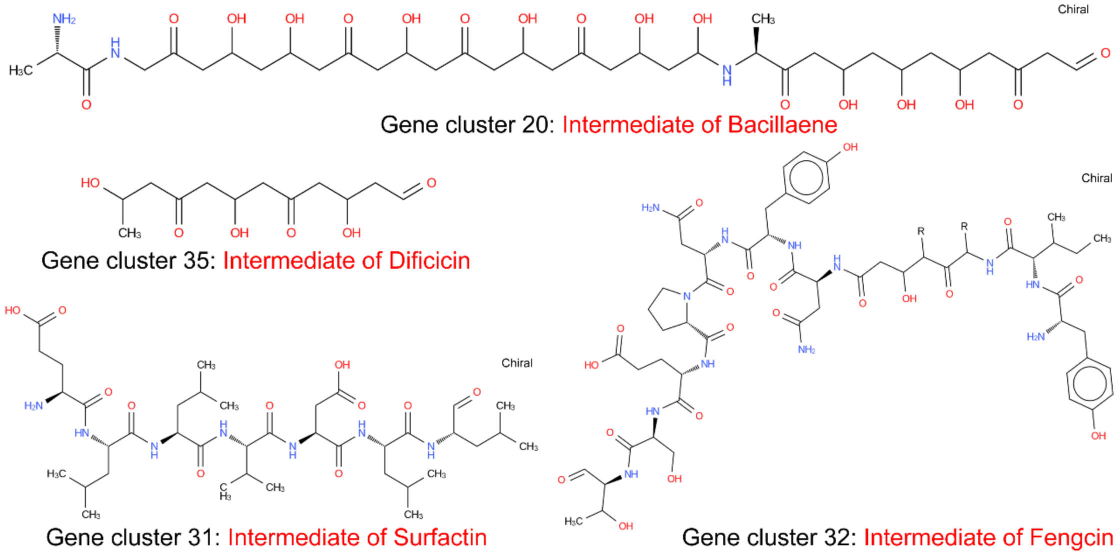

Figure S5. Predicted intermediate structure of antimicrobial compounds produced by UUS-1.

Table S1 Bioactivity of secondary metabolite clusters identified in the genome of *B. velezensis* strain UUS-1.

Compound	Synthase type	Scaffold	Size	Similarity gene	Bioactivity
**Butirosin**	OtherKs	scaffold1	41.2 kb	7 %	antibacterial
**Macrolactin**	Terpene	scaffold1	86.1 kb	100 %	antibacterial
**Bacilysin**	Other	scaffold2	41.4 kb	100 %	antibacterial
**Difficidin**	Transatpks	Scaffold3	19.0 kb	40 %	antibacterial
**Bacillibactin**	Bacteriocin-Nrps	Scaffold4	66.8 kb	100 %	siderophore
**Bacillaene**	Transatpks-Nrps	Scaffold5	122.7 kb	100 %	antibacterial
**Difficidin**	Transatpks	Scaffold6	46.0 kb	53 %	antibacterial
**Surfactin**	Nrps	Scaffold9	55.5 kb	82 %	multiple
**Fengcin**	Transatpks-Nrps	Scaffold10	88.1 kb	86 %	antifungal
**Plipastatin**	Nrps	Scaffold15	22.4 kb	46 %	antifungal
**Difficidin**	Transatpks	Scaffold16	23.8 kb	26 %	antibacterial
**Plipastatin**	Nrps	Scaffold18	22.4 kb	23 %	antifungal
**Fengcin**	Transatpks-Nrps	Scaffold19	9.8 kb	20 %	antifungal

Table S2 Contents and concentrated ratio of elements from seawater.

Element	Content (mg/Kg)				Concentrated Ratio		
	Seawater	Wet Bacteria	Dry Bacteria	Initial Culture	Wet Bacteria	Dry Bacteria	Initial Culture
**Ca**	413.36	1832.89	23731.21	2601305. 76	4. 43	57. 41	1290. 88
**Li**	0.134	0.27	3.54	388. 53	2. 03	26. 38	593. 22
**Cr**	0.07	14.64	189.61	20784. 69	220. 55	2855. 64	64209. 73
**Cu**	0.30 μg/Kg	2.27	29.48	3232. 37	7591. 85	98294. 56	2210173. 60
**Fe**	2.80 μg/Kg	96.05	1243.72	136330. 96	34307. 05	444186. 09	9987616. 20
**K**	456.93	6031.62	78093.68	8560269. 23	13. 20	170. 90	3842. 93
**Na**	8500.90	12499.34	161833.68	17739461. 54	1. 47	19. 037	428. 05
**Ni**	0.73 μg/Kg	5.52	71.54	7841. 94	7569. 16	98000. 72	2203566. 50
**U**	3.10 μg/Kg	16.51	213.89	9456. 59	5329. 01	68996. 77	625746. 40
**V**	1.20 μg/Kg	0.05	0.68	75. 05	44. 07	570. 60	12830. 07
**Zn**	0.08	18.18	235.42	25806. 58	237. 37	3073. 40	69106. 24
**Mg**	1132.90	2265.01	29325.94	3214575. 68	1. 99	25. 88	582. 04
**S**	0.80	1257.51	16281.47	1784700. 84	1579. 78	20454. 11	459914. 96
**Si**	0.72	2676.43	34652.78	3798478. 84	3698. 77	47889. 42	1076803. 70
**P**	0.02	4360.77	56460.52	6188942. 30	177990. 70	2304511. 27	51817413. 60
**Mn**	1.30 μg/Kg	26.66	345.24	37844. 07	20511. 69	265572. 46	5971452. 93
**B**	3.28	19.50	252.47	27675. 28	5. 94	77. 02	1731. 79
